# Patterns of change in treatment, response, and outcome in patients with follicular lymphoma over the last four decades: a single-center experience

**DOI:** 10.1038/s41408-020-0299-0

**Published:** 2020-03-05

**Authors:** Pablo Mozas, Ferran Nadeu, Alfredo Rivas-Delgado, Andrea Rivero, Marta Garrote, Olga Balagué, Blanca González-Farré, Luis Veloza, Tycho Baumann, Eva Giné, Julio Delgado, Neus Villamor, Elías Campo, Laura Magnano, Armando López-Guillermo

**Affiliations:** 1 Department of Hematology, Hospital Clínic, Barcelona, Spain; 2 Institut d’Investigacions Biomèdiques August Pi i Sunyer (IDIBAPS), Barcelona, Spain; 3 Centro de Investigación Biomédica en Red de Cáncer (CIBERONC), Barcelona, Spain; 4 Hematopathology Unit, Department of Pathology, Hospital Clínic, Barcelona, Spain; 5 University of Barcelona, Barcelona, Spain

**Keywords:** Lymphoma, Epidemiology

## Abstract

Although the introduction of immunotherapy has improved outcomes for follicular lymphoma (FL) patients, histological transformation (HT) and early relapse still confer a poor prognosis. We sought to describe the patterns of change in treatment, response, and outcome of FL patients at our institution over the last four decades. Seven hundred and twenty-seven patients (389 F/338 M; median age, 57 years) consecutively diagnosed with grade 1-3a FL between 1980 and 2017, categorized into four decades according to the time of diagnosis, constituted the study population. Clinical characteristics, treatment, response, absolute and relative survival, HT, second malignancies (SM), and causes of death were assessed. Median OS for the entire cohort was 17.6 years. From decade 1 to 4, there was an increase in the complete response rate (48 to 70%), progression-free survival (40 to 56% at 5 years), OS (77 to 86% at 5 years), and relative survival ratio (0.83 to 0.94 at 5 years), with no significant differences in the risk of HT or SM. Lymphoma remained the most common cause of death in all four decades. These findings illustrate the overall improvement in outcome for FL patients, but support the need for further research into risk stratification and management.

## Introduction

Follicular lymphoma (FL), the most common type of indolent B-cell lymphoma^1^, is characterized by a long survival, despite a pattern of continuous relapses during follow-up^2^, and shorter duration of responses in each relapse^3^. Although data initially indicated that survival of FL patients had remained unchanged during the last three decades of the 20th century^4^, more recent analyses have acknowledged that improvements in therapy, most likely related to the use of rituximab, as well as supportive measures, may have contributed to a better progression-free (PFS) and overall survival (OS) in recent years^5–15^. Despite a median OS now approaching 20 years, early progression (progression of disease within 24 months, or POD24), defined as a relapse within the first 2 years of frontline treatment, confers a much poorer prognosis^16^. Likewise, histologic transformation (HT) to a high-grade lymphoma significantly reduces survival^17,18^, although recent studies have suggested that its incidence might be lower in the rituximab era^19^. Some single-center and registry studies have described the changes in survival of FL patients over the last decades and trends in relative survival according to the general population^7,9^. However, to our knowledge, no single-center series has concurrently examined the trends in observed and relative survival, the risk of HT and second malignancies (SM), and the causes of death over a long period of time.

In this retrospective analysis of a single-center cohort, we sought to describe the patterns of change in baseline characteristics, treatment, response, PFS, absolute and relative OS, risk of HT and SM, and causes of death of FL patients over the last four decades at our institution.

## Methods

### Patients

Seven hundred and twenty-seven patients (389 females, 338 males; median age, 57 years) consecutively diagnosed with grade 1-3a FL at Hospital Clínic of Barcelona between January 1980 and December 2017 were included in the present study. All the available diagnostic specimens (most of them dating from 1990 onwards) underwent pathological review. Patients with grade 3b FL, primary gastrointestinal or cutaneous FL, or composite lymphoma were excluded. Baseline patient and disease characteristics, type of frontline treatment, PFS and OS, and risk of HT and SM were assessed retrospectively. Considering the time necessary for a complete diagnosis and staging, frontline treatment was defined as “immediate” if it was started within the first 2 months after diagnosis, or as a “watchful waiting” policy if it was deferred for a longer period of time. Treatment choices in each of the decades were in accordance with the policies of our Department. During decades 1 and 2, a “watchful waiting” strategy was undertaken only in patients 65 years old or older, while in decades 3 and 4, the initiation of treatment responded to the GELF criteria^20^.

Since our study cohort spans a 38-year period, we categorized patients into four groups according to the decade of diagnosis: decade 1, 1980–1989; decade 2, 1990–1999; decade 3, 2000–2009; and decade 4, 2010–2017. We decided to include the last period in the analyses, although, due to short follow-up, data concerning survival and the risk of HT and SM should be interpreted with caution. The study was conducted according to the Hospital Clínic Institutional Review Board and in accordance with the Declaration of Helsinki.

### Response assessment and outcome

In most cases diagnosed during decades 1 and 2, response to treatment was evaluated using only computed tomography (CT). With the advent of positron emission tomography (PET), PET-CT was increasingly used as a staging and response assessment tool during decades 3 and 4. Therefore, we use the term complete response unconfirmed (CRu) when there was a residual mass on the CT scan without fluorodeoxyglucose uptake on the PET study. For response assessment purposes, CR and CRu were considered equivalent. Definitions of complete response (CR), partial response (PR), and treatment failure were the standard^21^.

HT was defined based on histologic or cytological criteria. It is challenging to affirm that second malignancies (SM) are causally related to FL or its treatment (especially for younger patients followed for a long period of time). We define SM as all types of malignant tumors appearing after the diagnosis of FL (including non-melanoma skin cancer). Time from the diagnosis of FL to that of the second neoplasm was registered. In the event of the development of more than one additional neoplasm in the same patient, only the first one was considered for the analysis.

Since the definition of disease-related or unrelated death might be a matter of discussion, we considered the following causes of death: progressive FL, treatment-related toxicities, neoplasms different from FL, and other/unknown causes.

### Statistical analysis

PFS was calculated from the date of the first dose of frontline treatment to the date of progression (if a PR was achieved after frontline treatment) or relapse (if a CR/CRu was achieved after frontline treatment). Hence, early progressors (POD24) were defined as patients experiencing progressive disease in the first 24 months after the initiation of frontline treatment. This analysis only included patients who received treatment and had a follow-up of more than 2 years. All patients were included for OS analysis, which was calculated from the date of diagnosis to the date of last follow-up or death from any cause. Kaplan-Meier survival curves were plotted for the four decades and compared for statistical differences by use of the log-rank test. To compare the OS observed in our cohort with that of the general population, patients were matched by age and sex with Spanish individuals from the Human Mortality Database, which accounts for other causes of death and provides an estimate of the cause-specific survival through relative survival analysis (relsurv R package). The relative survival ratio (RSR), calculated by dividing the observed survival of our cohort by the expected survival of the general population, was intended to reflect the reduction in life expectancy due to lymphoma. To estimate the risk of HT and of SM, where competitive events are possible, cumulative incidence was calculated (cmprsk R package) and compared by use of Gray’s test^22^. Likewise, when causes of death were evaluated through cause-specific cumulative incidence of mortality, competing risk of death was used. The χ^2^ test or p-exact test were used for comparison of categorical variables among the four decades. Nonparametric tests were used when necessary. *P* values <0.05 were considered to indicate statistical significance.

## Results

### Patient characteristics

Baseline characteristics of the 727 patients are shown in Table 1. Patients were distributed according to the year of diagnosis: decade 1 (1980–1989), *n* = 79 (11%); decade 2 (1990–1999), *n* = 163 (22%); decade 3 (2000–2009), *n* = 254 (35%); and decade 4 (2010–2017), *n* = 231 (32%). Median follow-up for the entire series (calculated only for surviving patients) was 7.6 years, and 31.8, 20.7, 12, and 3.2 years for decades 1–4, respectively. Most features did not follow a significant trend over decades, except for the more advanced age at diagnosis and better performance status in decade 4 (*P* < 0.001).

**Table 1:** Baseline features and response to frontline treatment of the 727 patients of the series.

Characteristics	Entire cohort (*n* = 727)	Decade 1 (1980–1989) (*n* = 79)	Decade 2 (1990–1999) (*n* = 163)	Decade 3 (2000–2009) (*n* = 254)	Decade 4 (2010–2017) (*n* = 231)	*P* value
Age, median (range)	57 (23–93)	54 (24–85)	54 (24–88)	57 (23–93)	61 (26–91)	<0.001
Female sex (%)	389 (53)	33 (42)	92 (56)	138 (54)	126 (54)	NS
Histologic grade 1 or 2 (%)	419 (84)	NA	59 (89)	184 (83)	172 (83)	NS
ECOG ≥ 2 (%)	64 (9)	16 (21)	18 (11)	15 (6)	15 (7)	<0.001
Ann Arbor stage IV (%)	433 (60)	45 (57)	97 (62)	149 (59)	142 (62)	NS
Elevated LDH (%)	126 (19)	17 (25)	30 (22)	42 (19)	37 (17)	NS
Elevated β2- microglobulin (%)	241 (43)	4 (57)	45 (36)	80 (37)	112 (52)	0.005
High-risk FLIPI (%)	163 (25)	20 (30)	38 (27)	39 (17)	66 (29)	0.009
CR/CRu (%)	437 (64)	37 (48)	85 (53)	177 (73)	138 (70)	<0.001
PR (%)	186 (28)	33 (42)	57 (36)	45 (19)	51 (26)	
Refractory disease (%)	54 (8)	8 (10)	18 (11)	19 (8)	9 (4)	

Response data are calculated only on the 677 patients of the series who received any treatment. Data concerning β2-microglobulin levels should be interpreted with caution for decade 1, since only seven patients had available data.

*ECOG* Eastern Cooperative Oncology Group, *LDH* lactate dehydrogenase, *FLIPI* Follicular Lymphoma International Prognostic Index, *NA* not available, *NS* not statistically significant, *CRu* complete response unconfirmed, *PR* partial response.

### Treatment and response to therapy

Initial treatment strategies given to patients in the entire cohort and according to the decade of diagnosis are depicted in Supplementary Table 1. Second- and third-line treatments are detailed in Supplementary Tables 2 and 3. The proportion of patients who underwent an initial “watchful waiting” strategy was 3%, 3%, 13%, and 30% for decades 1 to 4, respectively (*P* < 0.001), with a median time of observation of 1.8 years (range, 3 months–9.3 years). Rituximab was part of the initial treatment in none of the patients in decades 1 and 2, and in 66 and 85% of patients in decades 3 and 4, respectively (*P* < 0.001). Of the patients treated with chemoimmunotherapy during decades 3 and 4, 15 and 60% received rituximab maintenance therapy, respectively. Cyclophosphamide, doxorubicin, vincristine, and prednisone (CHOP) were the most frequent frontline chemotherapy regimen used in decades 1 and 2 (64 and 75% of patients, respectively), while this regimen was only used in combination with immunotherapy in decade 4. The advent of the anti-CD20 antibody rituximab made its combination with CHOP (R-CHOP) the preferred regimen in decades 3 and 4 (39 and 47%, respectively). Response to treatment is detailed in Table 1. For the entire cohort, the percentage of patients achieving a CR/CRu and a PR after frontline treatment was 64 and 28%, respectively, while 8% of patients had refractory disease. A steady increase in the rate of CR/CRu was seen, being of 48%, 53%, 73%, and 70% for decades 1–4, respectively, with the corresponding decrease in the rate of PR and refractory cases. Interestingly, cases not responding to frontline therapy decreased from 10% in decade 1–4% in decade 4 (*P* < 0.001).

### Risk of relapse/progression,

**Fig. 1:**
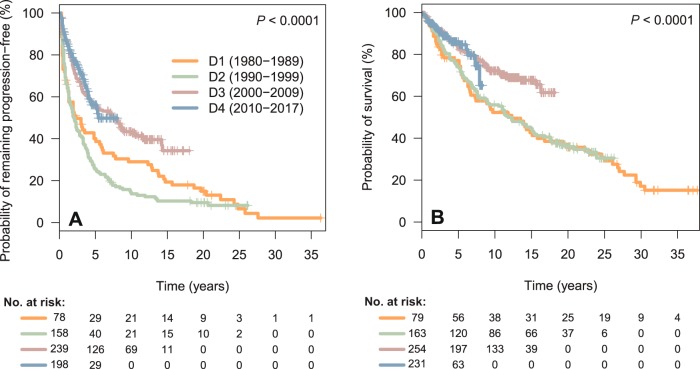
**a** progression-free survival of the 677 FL patients who received any treatment, according to the decade of diagnosis. **b** overall survival of the 727 FL patients of the series, according to the decade of diagnosis. D decade, FL follicular lymphoma.

Data concerning PFS, POD24, OS, risk of HT, and risk of SM are depicted in Table 2. Three hundred and sixty-four patients (54%) were refractory or experienced relapse/progression during follow-up, with a median PFS for the entire cohort of 4.3 years, and a 10-year PFS of 34%. A significant increase in PFS was observed over the decades, with a median PFS of 2.3, 2.1, 7.5 years, and unreached for decades 1 to 4, respectively (*P* < 0.001, Fig. 1a). In addition, the proportion of patients progressing within the first 2 years after the initiation of frontline therapy (early progressors or POD24) significantly decreased over the decades (44%, 46%, 25%, and 19% for decades 1–4, respectively, *P* < 0.001). When evaluating survival from an event-defining timepoint (the time of progression for POD24 patients, or 2 years of follow-up for non-POD24 patients), no significant differences were noted among decades, although early progression retained its negative impact on survival for patients diagnosed in all four decades (*P* < 0.0001; Supplementary Fig. 1).

**Table 2:** PFS, POD24, OS, risk of HT, and risk of SM of the 727 patients with follicular lymphoma.

	Entire cohort (*n* = 727)	Decade 1 (1980–1989) (*n* = 79)	Decade 2 (1990–1999) (*n* = 163)	Decade 3 (2000–2009) (*n* = 254)	Decade 4 (2010–2017) (*n* = 231)	*P* value
PFS						
Median PFS, years	4.3	2.3	2.1	7.5	NR	<0.001
5-year PFS, %	46	40	26	55	56	
10-year PFS, %	34	29	14	43	NR	
Early progression (POD24) (%)	204 (30)	34 (44)	73 (46)	60 (25)	37 (19)	<0.001
OS						
Median OS, years	17.6	12.3	11.8	NR	NR	<0.001
5-year OS, %	81	77	74	83	86	
10-year OS, %	65	52	56	72	–	
Histological transformation						
5-year risk, %	7	7	9	5	8	NS
10-year risk, %	10	8	15	7	–	
Second malignancy						
5-year risk, %	5	1	5	7	4	NS
10-year risk, %	10	5	9	12	–	

Due to short follow-up for Decade 4, data concerning survival and risk of histological transformation and second malignancies should be interpreted with caution.

*PFS* progression-free survival, *POD24* progression of disease within the first 24 months of frontline treatment initiation, *OS* overall survival, *NR* not reached, *NS* not statistically significant.

### Histological transformation and second malignancies

**Fig. 2:**
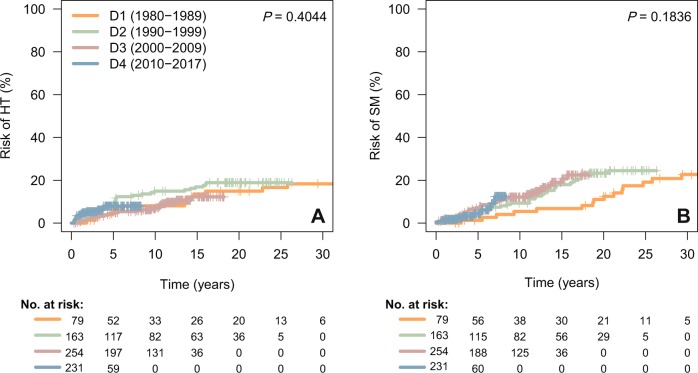
Risk of histological transformation (HT, **a**) and of second malignancies (SM, **b**). D decade.

Risk of HT and SM are plotted in Fig. 2 and Supplementary Fig. 2. HT to diffuse large B-cell lymphoma (DLBCL) was seen in 13 (17%), 30 (18%), 23 (9%), and 15 (7%) patients diagnosed during decades 1–4, respectively. For the entire series, the median time to HT was 4.1 years (range, 3 months–25.8 years). The 5- and 10-year risks of HT for the entire cohort were 7 and 10%, respectively, with no significant differences across decades. Likewise, we did not find significant differences in the rate of HT according to the frontline exposure of rituximab, with or without maintenance.

The diagnosis of a second malignancy after the diagnosis of FL was made in 16 (20%), 37 (23%), 39 (15%), and 10 (4%) patients for those diagnosed during decades 1–4, respectively. The median time to the development of a SM was 7.7 years (range, 1 month–33 years), with a 10-year risk of SM of 10% for all patients, and 5%, 9%, and 12% for decades 1–3, respectively, with no significant differences across decades. Hematological malignancies constituted the most frequent type of SM, which accounted for 20% of cases, including five cases of acute myeloid leukemia. Other frequent sites for the development of SM were the genitourinary tract (17%), the lung and ear, nose, and throat area (15%), and the skin (non-melanoma skin cancer, NMSC, 11%). When the cumulative incidence of SM was evaluated according to the site (hematological malignancies, solid tumors excluding NMSC, and NMSC alone), no significant differences were observed across decades. Likewise, we did not find significant differences in the risk of SM according to the treatment with fludarabine or stem cell transplantation (autologous or allogeneic).

### Overall and relative survival

Two hundred and sixty-eight patients (37%) eventually died during the time of follow-up. Median OS for the entire cohort was 17.6 years, and the 10-year OS rate was 65%. As seen for PFS, there was a significant increase in OS, the median OS being 12.3 and 11.8 years for decades 1 and 2, respectively, and unreached for decades 3 and 4 (*P* < 0.001, Fig. 1b). The 10-year OS rate for decades 1 to 4 was 52%, 56%, 72%, and not calculable, respectively.

**Fig. 3:**
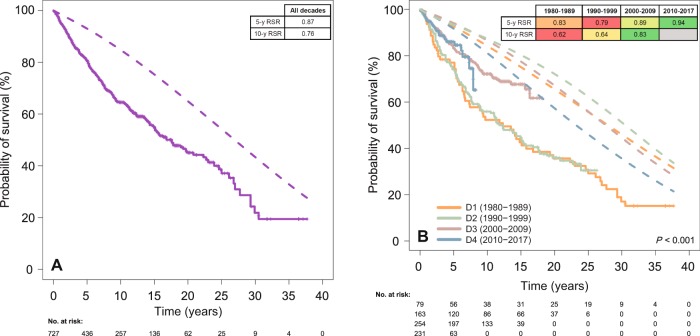
Relative survival ratios (RSR, calculated by dividing observed by expected survival) are detailed in the built-in tables and color-coded within each row, red and green representing poorer and better RSR values, respectively. **a** Comparison of observed (solid line) versus expected OS (dashed line) for the global series. **b** Comparison of observed (solid line) versus expected OS (dashed line) according to the decade of diagnosis. D decade, y year.

The outcome of patients along the decades was studied in terms of relative survival (RS) with respect to the general Spanish population (Fig. 3, Supplementary Table 4, and Supplementary Fig. 3). As mentioned above, PFS and OS greatly improved across the decades. This improvement was substantially more evident for FL patients than the gains in life expectancy for the sex- and age-matched general population. Importantly, however, the diagnosis of FL remained a burden for OS, which was significantly inferior to the expected survival of the general population. The 5-year relative survival ratios (RSR) for decades 1 to 4 were 0.83, 0.79, 0.89, and 0.94 respectively. The 10-year RSR were 0.62, 0.64, 0.83, and not calculable, for decades 1–4, respectively. When assessing the evolution of RS according to age (less than 50 years, 50–69 years, and 70 years or more) and sex, the trend of improvement was seen across decades for both sexes and all three age groups.

### Causes of death

The exact cause of death was known for 233 (95.2%) of the 268 deceased patients. For patients dying within the 10 years of the diagnosis of FL, 61% of those diagnosed in decade 1 died due to progression of FL, while this percentage progressively decreased during the last two decades (42 and 45% for decades 3 and 4, respectively, *P* = 0.020, Table 3). Consequently, the percentage of patients dying from other/unknown causes increased (28 and 35% for decades 1 and 4, respectively).

**Table 3:** Causes of death in the 204 patients who died within the first 10 years after diagnosis.

	Entire cohort (*n* = 204)	Decade 1 (1980–1989) (*n* = 36)	Decade 2 (1990–1999) (*n* = 71)	Decade 3 (2000–2009) (*n* = 66)	Decade 4 (2010–2017) (*n* = 31)
Progression of FL (%)	111 (54)	22 (61)	47 (66)	28 (42)	14 (45)
Complications of therapy (%)	18 (9)	1 (3)	5 (7)	9 (14)	3 (10)
Other neoplasms (%)	31 (15)	3 (8)	9 (13)	16 (24)	3 (10)
Others/unknown (%)	44 (22)	10 (28)	10 (14)	13 (20)	11 (35)

The Others/unknown group is composed of 23 patients (11%) dying of other causes (mainly geriatric complications and cardiovascular disease), and 21 patients (10%) for whom information concerning the exact cause of death was not available.

*FL* follicular lymphoma.

**Fig. 4:**
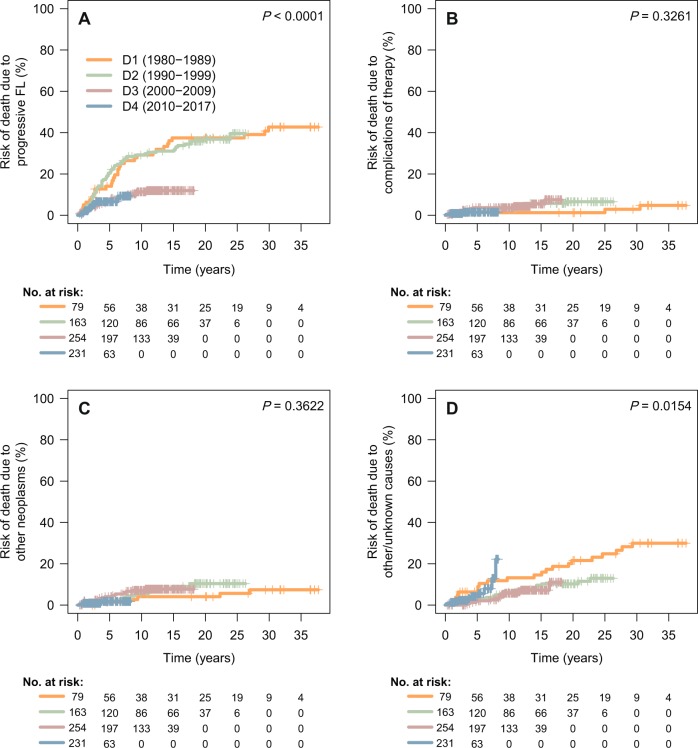
Cause-specific cumulative incidence of mortality, using competing risk of death. **a** death due to progressive FL; **b** death due to complications of therapy; **c** death due to other neoplasms; **d** death due to other/unknown causes.

Using cumulative incidence of mortality with competing risk of death, 5-year mortality due to progressive FL was estimated at 14 and 20% for decades 1 and 2, respectively, in contrast with 7 and 7% for decades 3 and 4, respectively (*P* < 0.0001). Cumulative incidence of death from complications of therapy or that due to other neoplasms was not significantly different across decades (Fig. 4 and Supplementary Table 5).

## Discussion

In the analysis of our cohort of 727 FL patients, spanning almost four decades, we sought to describe variations in care and outcome. Patients diagnosed during decade 4 were older but had a better performance status. These trends presumably reflect changes in the reference population of our institution, and the performance of a greater number of diagnostic tests in otherwise asymptomatic elderly individuals, which might recognize FL at an older age. No patient received immunotherapy during decades 1 and 2, being CHOP-like regimens the most common therapeutic options. With the advent of rituximab, R-CHOP was the most used first line treatment (around 40% of patients in decades 3 and 4).

The fraction of patients achieving a CR after frontline treatment markedly increased over time, likely on account of the adoption of immunotherapy. However, taking into account that PET-CT can help consider as a CR a study where there is a residual mass without metabolic activity, the advent of this technique cannot be discarded as a contributing factor to an improvement in observed responses. This deepening of responses translated into a better PFS (40–56% at 5 years, for decades 1 and 4, respectively) and OS (77–86% at 5 years, for decades 1 and 4, respectively), as previously shown by multicenter studies in the rituximab era^23^.

Evidences of improvement in PFS and OS in patients with FL over the last decades have been published in recent years. Some single-center and registry studies have shown an improved survival of FL patients with respect to a sex- and age-matched population^7,9^. Our series shows the same trend, with a 5-year RSR increasing from 0.83 in decade 1 to 0.94 in decade 4 and a 10-year RSR increasing from 0.62 in decade 1 to 0.83 in decade 3. To evaluate the causes of death in the present series, we first considered only patients dying within the first decade after diagnosis, with the intention of equalizing follow-up among decades. Although the proportion of patients dying due to progressive FL has decreased across decades (61–45% from decades 1–4), most patients still die from lymphoma-related causes (55% in decade 4, considering progressive disease and therapy-related complications), in line with recent results arising from large cohorts^24^. This fact underscores the need for new therapies with improved toxicity profiles and a lower potential to induce SM. When evaluating causes of death of the entire series using a cumulative mortality risk analysis with competing causes of death, we were able to identify a marked reduction in the cumulative incidence of death due to progressive disease.

In contrast with recent reports describing a reduced risk of HT in the rituximab era^19^, we were not able to identify significant differences in its incidence among decades, presumably to the relatively small number of this type of event in each of the decades. However, HT rates in the last two decades of the study are superimposable to that reported in the Aristotle study^19^.

The appearance of a SM represents one of the concerns of the use of cytotoxic agents, which has led to a growing interest in the development of chemotherapy-free regimens. In the rituximab era, its cumulative incidence has been estimated to be of 2.9% at 10 years for SM arising at any site^24^, and of 2.7% at 10 years for second hematological malignancies specifically^25^. In our series, the 10-year incidence of SM of any site was 10%, with no significant differences among decades, and hematological malignancies were the most common subtype. Although follow-up is short, the incidence of SM observed in decade 4 strongly compares with that reported by Sarkozy and colleagues^24^.

We have to acknowledge some limitations of our study. First, due to short follow-up of patients in decade 4, data concerning survival and the risk of HT and SM should be interpreted with caution. Second, baseline characteristics were not totally uniform among decades. However, the fact that patients in decade 4 were older might further underscore the improvement in outcomes of FL patients, since older individuals would be expected to fare more poorly. Third, due to historical reasons, the number of patients in decade 1 was lower, as was the availability of a diagnostic specimen for histological review, and data regarding the cause of death, which might explain the higher percentage of patients dying from other/unknown causes in this period of time. Fourth, the low number of patients developing a HT in each decade might hamper the ability to observe a decreased HT rate in the rituximab era. Finally, considering the nature of this disease, an analysis of second and subsequent lines of therapy might help to better characterize progression/relapse patterns and outcomes. Among the strengths of the current study, however, are the large number of patients from a single institution, diagnosed and treated with comparable standards within each decade, the high availability of clinical characteristics and follow-up data, and the use of robust statistical methods to analyze relative survival and competing risks (HT, SM, and causes of death).

In conclusion, in our series, we observed that FL patients have experienced an increase in the CR rate after frontline treatment, with a positive impact on PFS, OS, RS, and cumulative incidence of death due to progressive FL. Conversely, the risk of HT and SM, as well as the percentage of lymphoma-related deaths have remained essentially unchanged, highlighting the need for further advances in prognostic and therapeutic tools for this disease.

## Supplementary information

Supplementary Figure 1

Supplementary Figure 2

Supplementary Figure 3

Supplementary Table 1

Supplementary Table 2

Supplementary Table 3

Supplementary Table 4

Supplementary Table 5
