# Supplementary material for: Patterns of change in treatment, response, and outcome in patients with follicular lymphoma over the last four decades: a single-center experience
Source: Blood Cancer J. 2020 Mar 5;10(3):31. doi: 10.1038/s41408-020-0299-0 (PMC7058022; doi:10.1038/s41408-020-0299-0)
Supplement: Supplementary file 1 — Supplementary Figure 1 [file 41408_2020_299_MOESM1_ESM.pdf]

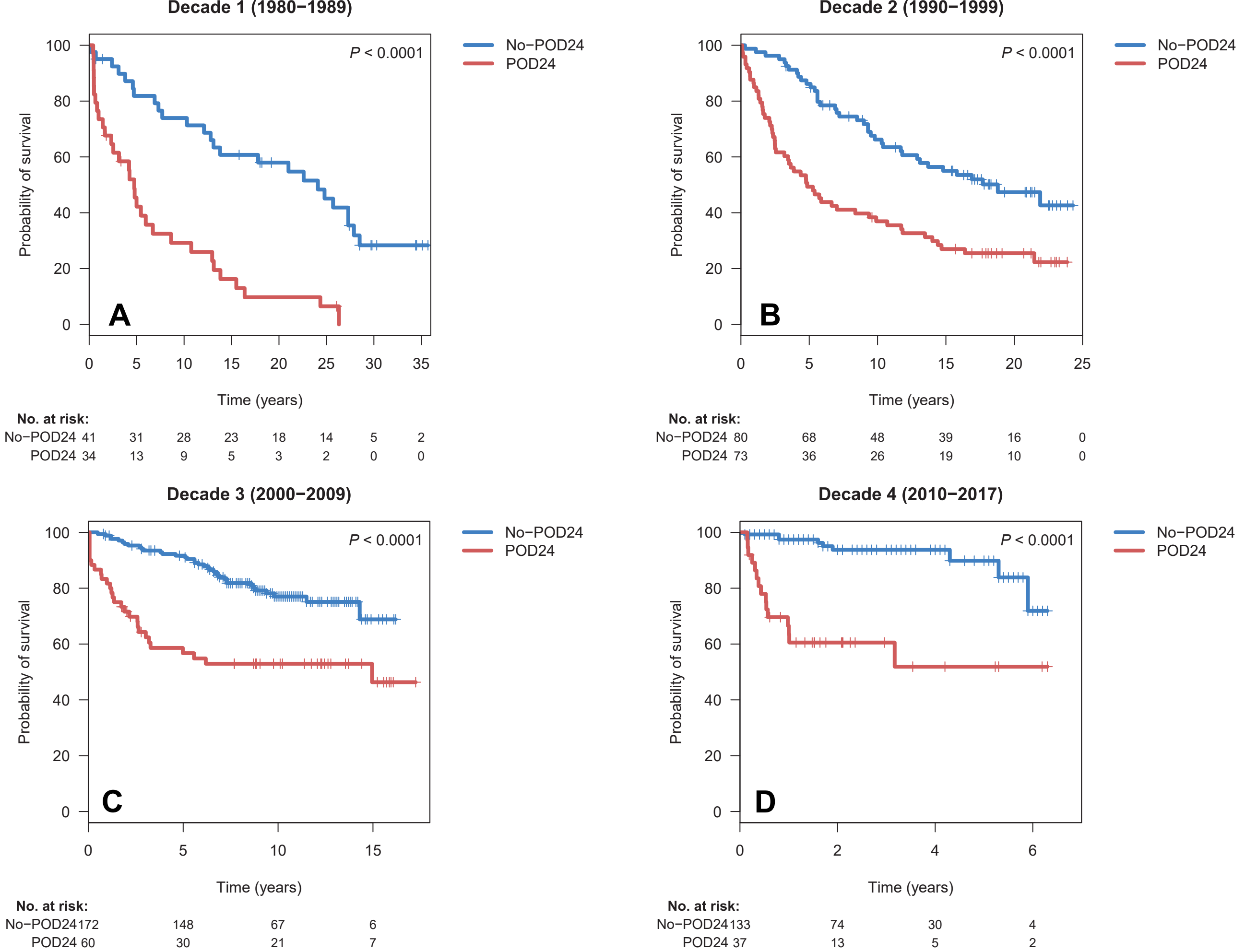

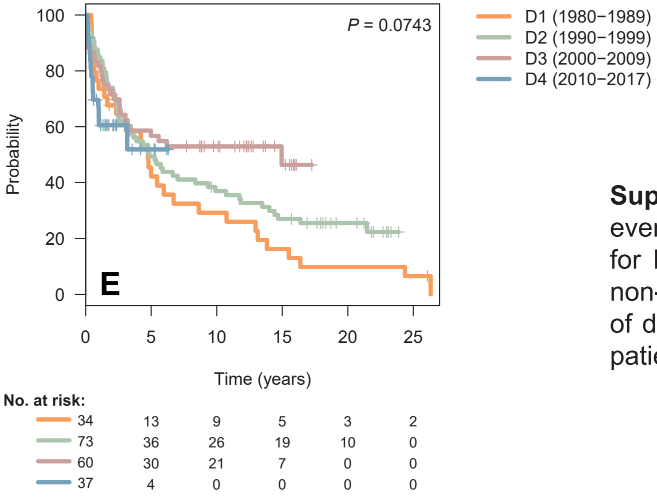

Supplementary Figure 1. Survival from an event-defining timepoint (the time of progression for POD24 patients, or 2 years of follow-up for non-POD24 patients), according to the decade of diagnosis (A through D), and only for POD24 patients (E).
