# Supplementary material for: Patterns of change in treatment, response, and outcome in patients with follicular lymphoma over the last four decades: a single-center experience
Source: Blood Cancer J. 2020 Mar 5;10(3):31. doi: 10.1038/s41408-020-0299-0 (PMC7058022; doi:10.1038/s41408-020-0299-0)
Supplement: Supplementary file 2 — Supplementary Figure 2 [file 41408_2020_299_MOESM2_ESM.pdf]

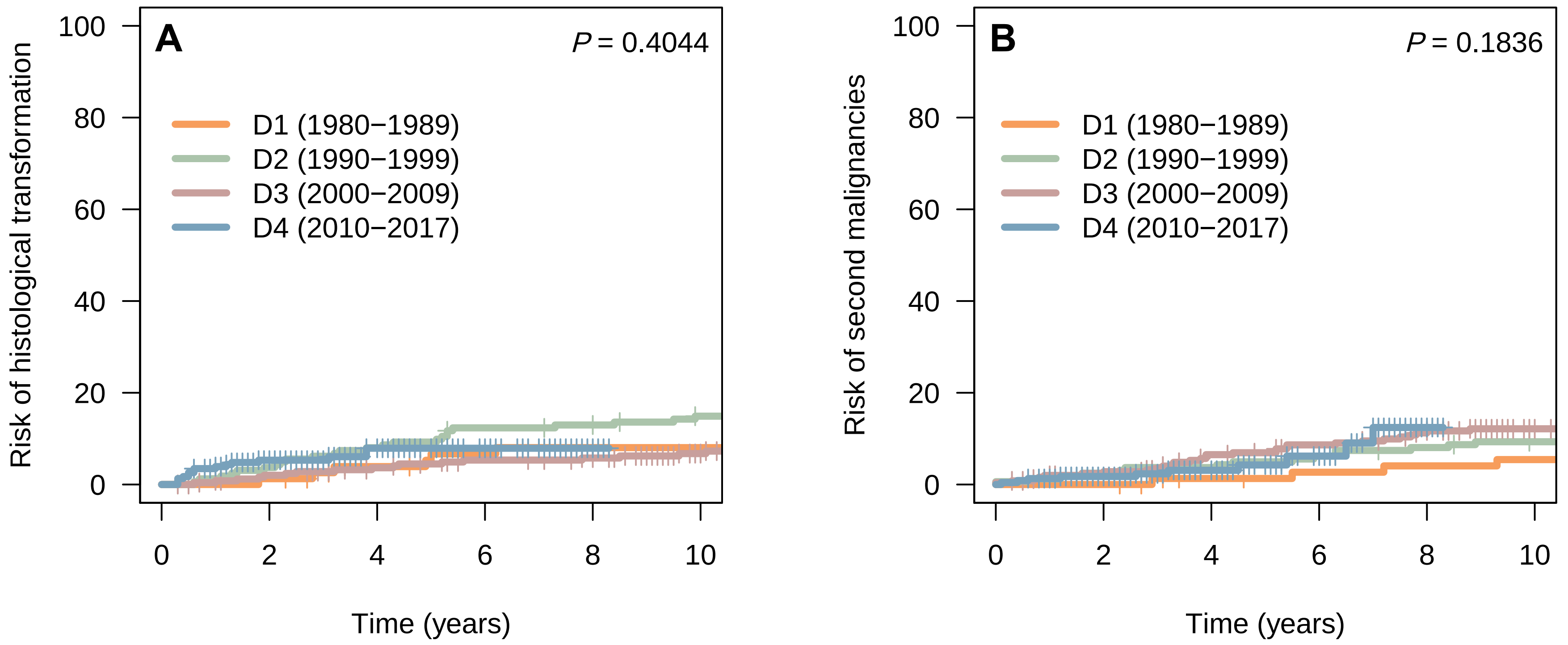

No. at risk:

D1 (1980−1989)	79	67	55	44	37	33
D2 (1990−1999)	163	143	124	106	92	82
D3 (2000−2009)	254	227	205	185	173	131
D4 (2010−2017)	231	175	85	35	5	0

No. at risk:

D1 (1980−1989)	79	68	58	48	42	38
D2 (1990−1999)	163	144	123	104	89	82
D3 (2000−2009)	254	229	200	178	165	125
D4 (2010−2017)	231	180	87	34	5	0

Supplementary Figure 2. Risk of histological transformation (A) and of second malignancies (B) with a follow-up limited to 10 years, according to the decade of diagnosis.
