# Supplementary material for: Patterns of change in treatment, response, and outcome in patients with follicular lymphoma over the last four decades: a single-center experience
Source: Blood Cancer J. 2020 Mar 5;10(3):31. doi: 10.1038/s41408-020-0299-0 (PMC7058022; doi:10.1038/s41408-020-0299-0)

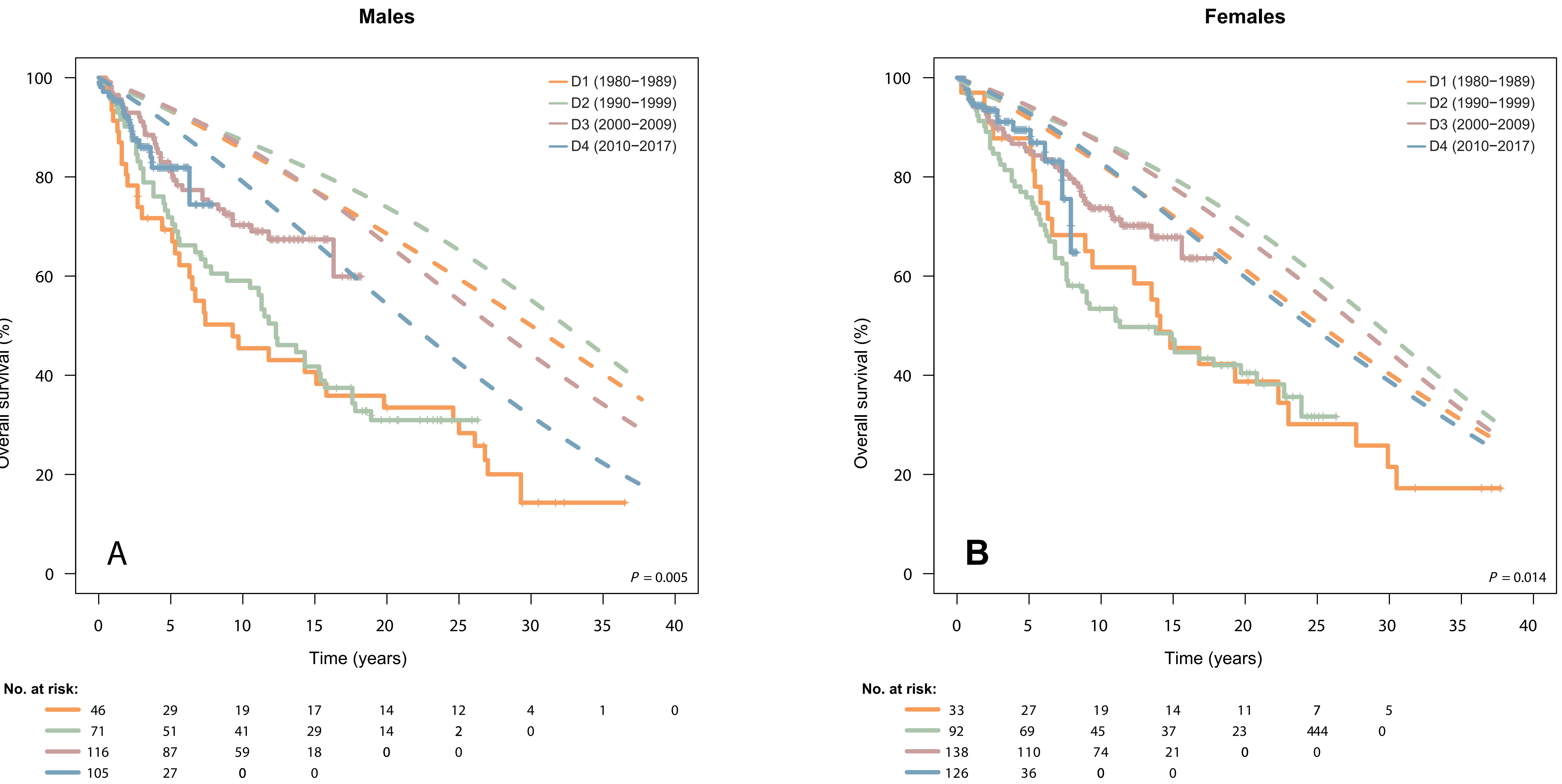

Supplementary Figure 3. Comparison of observed (solid line) versus expected (dashed line) OS, according to the decade of diagnosis. A: male patients only; B: female patients only; C: patients younger than 50 years; D: patients between 50 and 69 years; and E: patients of 70 years or older.

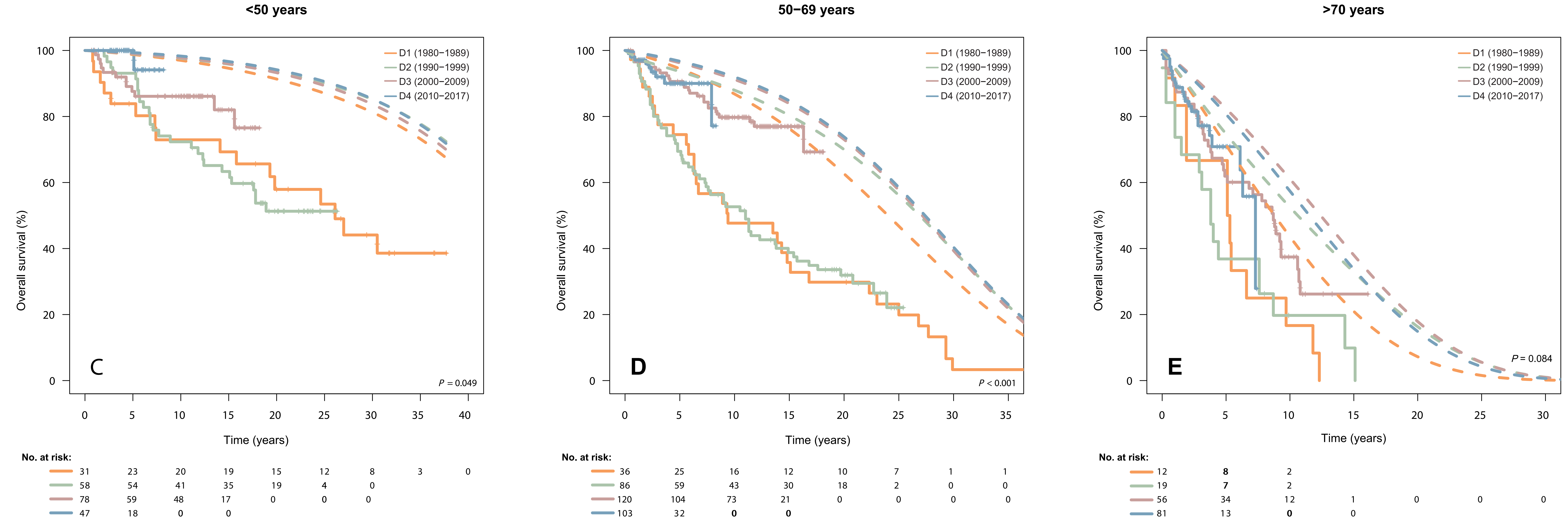

Supplement: Supplementary file 3 — Supplementary Figure 3 [file 41408_2020_299_MOESM3_ESM.pdf]
