# Supplementary material for: Patterns of change in treatment, response, and outcome in patients with follicular lymphoma over the last four decades: a single-center experience
Source: Blood Cancer J. 2020 Mar 5;10(3):31. doi: 10.1038/s41408-020-0299-0 (PMC7058022; doi:10.1038/s41408-020-0299-0)
Supplement: Supplementary file 4 — Supplementary Table 1 [file 41408_2020_299_MOESM4_ESM.pdf]

SUPPLEMENTARY MATERIAL

Supplementary Table 1. Initial treatment strategies.

**Treatment**	**Entire cohort (N=727)**	**Decade 1 (1980-1989) (N=79)**	**Decade 2 (1990-1999) (N=163)**	**Decade 3 (2000-2009) (N=254)**	**Decade 4 (2010-2017) (N=231)**
Watchful waiting (%)	110 (15)	2 (3)	5 (3)	34 (13)	69 (30)
R-CHOP (%)	174 (24)	0	0	**92 (36)**	**82 (35)**
R-CVP (%)	43 (6)	0	0	25 (10)	18 (8)
Other R-containing regimens (%)	45 (6)	0	0	25 (10)	20 (9)
Single-agent rituximab (%)	48 (7)	0	0	8 (3)	40 (17)
CHOP-like (%)	199 (27)	**50 (63)**	**122 (75)**	27 (11)	0
Fludarabine-based (%)	34 (5)	0	3 (2)	31 (12)	0
Other treatments/unknown (%)	74 (10)	27 (34)	33 (20)	12 (5)	2 (1)

Most used treatment modality in each decade is highlighted in bold.

CVP, cyclophosphamide, vincristine, and prednisone; CHOP, cyclophosphamide, doxorubicin, vincristine, and prednisone; R, rituximab.
