# Supplementary material for: Patterns of change in treatment, response, and outcome in patients with follicular lymphoma over the last four decades: a single-center experience
Source: Blood Cancer J. 2020 Mar 5;10(3):31. doi: 10.1038/s41408-020-0299-0 (PMC7058022; doi:10.1038/s41408-020-0299-0)
Supplement: Supplementary file 5 — Supplementary Table 2 [file 41408_2020_299_MOESM5_ESM.pdf]

**Supplementary Table 2.** Second-line treatment strategies

| Second-line treatment | | 1980-1989 | 1990-1999 | 2000-2009 | 2010-2017 | Total |
|---|---|---|---|---|---|---|
| **No treatment** | n | 2 | 2 | 6 | 5 | 15 |
| | % | 4% | 2% | 6% | 10% | 4% |
| **R-CHOP/R-CVP** | n | 1 | 4 | 15 | 5 | 25 |
| | % | 2% | 3% | 14% | 10% | 7% |
| **Single-agent rituximab** | n | 3 | 8 | 25 | 2 | 38 |
| | % | 5% | 6% | 23% | 4% | 11% |
| **Benda/Fluda-based (+/- R)** | n | 1 | 34 | 19 | 15 | 69 |
| | % | 2% | 26% | 17% | 29% | 20% |
| **Other chemo regimens** | n | 25 | 40 | 17 | 16 | 98 |
| | % | 45% | 31% | 16% | 31% | 28% |
| **Chlorambucil and other agents** | n | 23 | 15 | 1 | 0 | 39 |
| | % | 41% | 12% | 1% | 0% | 11% |
| **ASCT** | n | 1 | 23 | 22 | 7 | 53 |
| | % | 2% | 18% | 20% | 14% | 15% |
| **Allo-SCT** | n | 0 | 3 | 4 | 1 | 8 |
| | % | 0% | 2% | 4% | 2% | 2% |

CVP, cyclophosphamide, vincristine, and prednisone; CHOP, cyclophosphamide, doxorubicin, vincristine, and prednisone; R, rituximab; Benda, bendamustine; Fluda, fludarabine; chemo, chemotherapy; ASCT, autologous stem cell transplantation; Allo-SCT, allogeneic stem cell transplantation.
