# Supplementary material for: Patterns of change in treatment, response, and outcome in patients with follicular lymphoma over the last four decades: a single-center experience
Source: Blood Cancer J. 2020 Mar 5;10(3):31. doi: 10.1038/s41408-020-0299-0 (PMC7058022; doi:10.1038/s41408-020-0299-0)
Supplement: Supplementary file 6 — Supplementary Table 3 [file 41408_2020_299_MOESM6_ESM.pdf]

Supplementary Table 3. Third-line treatment strategies

Third-line treatment		1980-1989	1990-1999	2000-2009	2010-2017	Total
**No treatment**	n	0	1	4	0	5
	%	0%	1%	6%	0%	3%
**R-CHOP/R-CVP**	n	1	7	6	0	14
	%	3%	9%	10%	0%	7%
**Single-agent rituximab**	n	2	6	8	1	17
	%	6%	7%	13%	7%	9%
**Benda/Fluda-based (+/- R)**	n	1	15	9	2	27
	%	3%	18%	15%	13%	14%
**Other chemo regimens**	n	18	30	16	12	76
	%	53%	37%	26%	80%	39%
**Chlorambucil and other agents**	n	8	1	0	0	9
	%	24%	1%	0%	0%	5%
**ASCT**	n	4	18	11	0	33
	%	12%	22%	18%	0%	17%
**Allo-SCT**	n	0	4	8	0	12
	%	0%	5%	13%	0%	6%

CVP, cyclophosphamide, vincristine, and prednisone; CHOP, cyclophosphamide, doxorubicin, vincristine, and prednisone; R, rituximab; Benda, bendamustine; Fluda, fludarabine; chemo, chemotherapy; ASCT, autologous stem cell transplantation; Allo-SCT, allogeneic stem cell transplantation.
