# Supplementary material for: Patterns of change in treatment, response, and outcome in patients with follicular lymphoma over the last four decades: a single-center experience
Source: Blood Cancer J. 2020 Mar 5;10(3):31. doi: 10.1038/s41408-020-0299-0 (PMC7058022; doi:10.1038/s41408-020-0299-0)
Supplement: Supplementary file 7 — Supplementary Table 4 [file 41408_2020_299_MOESM7_ESM.pdf]

Supplementary Table 4. 5- and 10-year survival (observed, expected, and relative survival ratio, RSR) for all patients, and according to the decade of diagnosis, sex, and age at diagnosis. RSR are color-coded within each row, red and green representing poorer and better RSR values, respectively.

			5-y OS				
			**All decades**	**1980-1989**	**1990-1999**	**2000-2009**	**2010-2017**
**All patients**		Series	80.7	77.0	74.1	83.3	86.0
		Pop	93.0	92.7	93.3	94.1	91.6
		RSR	0.87	0.83	0.79	0.89	0.94
**Sex**	**Male**	Series	77.0	69.4	71.8	81.1	81.9
		Pop	92.5	93.3	93.4	93.8	90.3
		RSR	0.83	0.74	0.77	0.86	0.91
	**Female**	Series	84.0	87.8	75.9	85.1	89.4
		Pop	93.4	91.8	93.3	94.3	92.8
		RSR	0.90	0.96	0.81	0.90	0.96
**Age**	**<50 y**	Series	91.0	83.9	93.1	87.6	94.1
		Pop	99.1	98.8	99.0	99.0	99.3
		RSR	0.92	0.85	0.94	0.88	0.95
	**50-69 y**	Series	82.7	74.5	69.4	90.6	90.0
		Pop	95.6	94.5	93.6	96.3	96.7
		RSR	0.87	0.79	0.74	0.94	0.93
	**70+ y**	Series	61.3	66.7	36.8	61.9	70.9
		Pop	79.9	71.4	74.8	82.4	80.7
		RSR	0.77	0.93	0.49	0.75	0.88

			10-y OS				
			**All decades**	**1980-1989**	**1990-1999**	**2000-2009**	**2010-2017**
**All patients**		Series	64.5	52.3	55.9	72.1	
		Pop	84.8	84.3	87.3	86.6	81.2
		RSR	0.76	0.62	0.64	0.83	
**Sex**	**Male**	Series	63.0	45.4	59.1	70.3	
		Pop	84.2	85.6	87.4	86.3	79.0
		RSR	0.75	0.53	0.68	0.81	
	**Female**	Series	65.8	61.8	53.4	73.7	
		Pop	85.3	82.4	87.2	86.8	82.9
		RSR	0.77	0.75	0.61	0.85	
**Age**	**<50 y**	Series	80.2	72.9	72.3	86.1	
		Pop	97.7	97.0	97.6	97.8	98.3
		RSR	0.82	0.75	0.74	0.88	
	**50-69 y**	Series	67.2	47.7	52.7	79.7	
		Pop	90.2	86.8	88.0	91.2	92.0
		RSR	0.75	0.55	0.60	0.87	
	**70+ y**	Series	31.5	16.7	19.7	37.4	
		Pop	57.1	43.6	52.6	61.1	57.4
		RSR	0.55	0.38	0.37	0.61	

y, year; OS, overall survival, Pop, population; RSR, relative survival risk. OS from the Series and the Population are expressed in %.
