# Supplementary material for: Patterns of change in treatment, response, and outcome in patients with follicular lymphoma over the last four decades: a single-center experience
Source: Blood Cancer J. 2020 Mar 5;10(3):31. doi: 10.1038/s41408-020-0299-0 (PMC7058022; doi:10.1038/s41408-020-0299-0)
Supplement: Supplementary file 8 — Supplementary Table 5 [file 41408_2020_299_MOESM8_ESM.pdf]

Supplementary Table 5. Cause-specific cumulative incidence of mortality, using competing risk of death, for all deceased patients.

	**Entire cohort (N=204)**	**Decade 1 (1980-1989) (N=36)**	**Decade 2 (1990-1999) (N=71)**	**Decade 3 (2000-2009) (N=66)**	**Decade 4 (2010-2017) (N=31)**	***P* value**
**Death due to progressive FL**						<0.0001
5-year risk, %	11	14	20	7	7	
10-year risk, %	19	29	29	12	-	
**Death due to complications of therapy**						NS
5-year risk, %	2	1	1	4	2	
10-year risk, %	3	1	3	4	-	
**Death due to other neoplasms**						NS
5-year risk, %	3	1	2	4	2	
10-year risk, %	6	4	6	7	-	

FL, follicular lymphoma.
